# Design of a Novel Intervention Model to Address Cardiovascular Health Disparities in the Rural Underserved Community of Phillips County Arkansas

**DOI:** 10.1089/heq.2021.0175

**Published:** 2022-03-15

**Authors:** Jessica W. Barnes, Mark Massing, Sushma Dugyala, Naomi Cottoms, Irion W. Pursell

**Affiliations:** ^1^ Division of Cardiology, Department of Medicine, University of Arkansas for Medical Sciences, Little Rock, Arkansas, USA.; ^2^ Tri County Rural Health Network Helena, West Helena, Arkansas, USA.

**Keywords:** community health worker, cardiovascular disease, social isolation, health disparities, health equity, racial equity

## Abstract

Devastating health-related disparities driven by an entanglement of factors disproportionately impact the underserved, low-wealth, and minority community of Phillips county (PC) in the Arkansas Delta Region (ADR). Cardiovascular disease continues to increase with widespread consequences on the local economy, health care systems, and population. Health care and community-based systems have been unsuccessful in reducing out-of-hospital cardiac death, particularly in the ADR, for many reasons. Herein, we share the strategy behind, planning, and goals of The Arkansas Lincoln Project, a novel neighborhood-based strategy bridging the gap between residents, social resources, and health care services in PC.

## Rural Arkansas Delta Challenges

Despite improvements in cardiovascular disease (CVD) management over the past 50 years, CVD-related disparities continue to disproportionately impact underserved low-wealth communities^1–7^ driven by a complex web of social, cultural, and medical influences.^8–10^ Nowhere is this more evident than in Phillips county (PC) in the Arkansas Delta Region (ADR), a community rich in African American history and culture. However, it also has a darker history of racial conflict including the 1919 Elaine Massacre, the worst racial massacre in United States history.^11^

Today economic prosperity in PC remains elusive due to struggles providing the infrastructure, skilled workforce, quality of life, and good paying jobs needed to maintain and grow the local economy, and enabling households to generate enough income to support their families. Overshadowing economic challenges of this majority African American community where >35% of residents live in poverty^12^ are devastating health disparities highlighted by the worst health factor and health outcome rankings of all 75 counties in the state^13^ and a CVD-related death rate of citizens <75 years old twice that of the national average.^12,14^

Out-of-hospital cardiac arrest (OHCA) is a devastating event that disproportionately affects the population in the ADR, and PC in particular^12–14^ (Table 1). The root causes of health disparities, including OHCA, are often the result of how resources are distributed among different groups. Those living in deprived areas such as PC and the ADR are likely to experience fewer of the positive benefits that communities can offer. To have the greatest impact on reducing inequality, specific deprived communities must be prioritized for support and prevention measures deployed that meet the needs of the target community.

**Table 1.** Phillips County Characteristics

	Number of residents per mi^2^	Median household income ($)	Residents living in poverty (%)	CVD death rate (per 100,000)	Health outcome 2021 ranking	Health factor 2021 ranking
Phillips County	23.8	$29,320	33.3%	139.4	75	75
ADR Overall	38.9	$39,463	23.6%	124.4	N/A	N/A
National Average	56.0	$65,712	16.2%	79.3	N/A	N/A

ADR, residents per mi^2^ from 2020 census data.^12^ Median household income 2019 data.^13^ CVD death rate from 2017 to 2019 data, both genders, all ethnicities, <75 years of age.^4,13^ Health outcome and factor ranking 1 is best, 75 is worst score among 75 Arkansas counties.

ADR, Arkansas Delta Region; CVD, cardiovascular disease.

Over the years, many health improvement projects, led by dedicated hard-working people, have been implemented in the ADR. Unfortunately, most of these projects were unsuccessful for a number of reasons including the traditional health care system being unable or unwilling to develop and maintain community-level interventions targeting communities with poor health outcomes, and grossly underfunded public health departments presiding over a cadre of fragmented programs that proved minimally effective.^4,5^

With understanding of previous shortcomings and challenges, The Arkansas Lincoln Project (TLP), a novel engagement and intervention model using a geospatial targeting strategy was developed over the past decade to focus on underserved low-wealth communities with health outcome disparities that address previous flaws preventing the rebalancing of rural health equity. TLP focuses on building a community health worker (CHW)–client relationship using a holistic approach that equally values primary disease prevention and acute medical care. Our goal is a successful OHCA prevention strategy prioritizing community engagement, access to safe and effective CVD risk reduction, and long-term focus on overall well-being in underserved communities that will yield measurable results.

## A Holistic Model

**FIG. 1.**
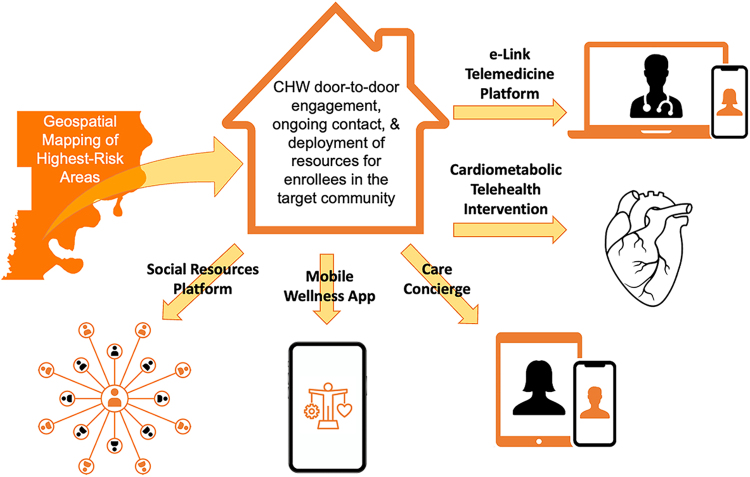
The Arkansas Lincoln Project is a coalition of local, county, state, and private key stakeholders mobilized through CHW-led neighborhood-based strategy in rural Phillips County in the Arkansas Delta Region. CHW, community health worker.

TLP is a coalition of local, county, state, and private key stakeholders mobilized through a CHW-led neighborhood-based strategy, bridging the gap between social resources and health care services and the rural ADR communities they serve (Fig. 1). This team is deployed using a geospatial approach, inspired by the American Red Cross Fire Prevention Program (FPP),^15–17^ where door-to-door efforts to replace smoke alarm batteries, or install a smoke alarm in residences without one, in communities with high levels of residential fires dramatically reduced home fires and deaths. Using a similar strategy to the FPP, we used premature natural death to identify highest risk PC neighborhoods to assess and address the needs of residents in each household of the target area (census tracts in PC).

TLP CHWs form the interface between health care systems and communities to identify and address social needs, improve health care access, quality of care, and cultural competence. In each geospatially identified highest risk PC neighborhood, TLP CHWs engage each resident over the age of 17 years with a holistic approach including a portfolio of programs and resources that address and manage social and medical factors leading to OHCA and populate a “living registry” with data enabling examination of TLP's impact on social and medical variables and key metrics including OHCA outcomes in the target neighborhoods.

TLP resident enrollees are offered access to a portfolio of services that currently include: (1) the 2-month obesity and CVD-risk reduction program administered by TLP partner 20Lighter,^18–21^ (2) a care concierge administered by TLP partner HeyRenee, and (3) the Health Science Index (HSI) wellness optimization mobile app. Enrollees are also offered referrals to social services and where indicated, telemedicine visits with health care providers. By establishing rapport, TLP CHWs build meaningful connections with socially isolated PC residents bringing access to interventions tailored to individual social and health needs with the goal of improving health and wellness, reducing CVD morbidity^22^ and mortality including OHCA,^8,23^ and ultimately increasing health equity.

The benefit of the TLP approach is that it does not limit the study population to a subjectively defined group of patients in PC who have suffered a cardiac arrest. Rather, this risk-based targeting approach incorporates a prospective design by recruiting individuals from an area with historically high rates of out-of-hospital premature natural death, collecting medical and social data, deploying strategies to directly impact modifiable CVD risk factors and improve wellness, and meeting enrollees' social needs and linking individuals to medical care. TLP's structure, flexibility, and focus on local resources allow adaptation within each neighborhood as needs evolve over time, enabling and encouraging the evolution from individual behavior change to long-term community prevention.

## Implementation of TLP in PC

In mid 2021, the TLP established door-to-door community engagement efforts in PC. This initial proof-of-concept engagement period is critical to inform a broader understanding of local communication needs enabling future community-driven identification of challenges and establishment of long-lasting complex relationships that allow long-term overall transformation of the local health system and improvement of health equity. TLP team leverages extensive expertise in developing community intervention strategies, a successful track record of community outreach and significant interest and enthusiasm of local and regional stakeholders for meaningful change in not only PC, but also in the 75% of ADR counties where minority populations and health disparities are significantly higher than the state average.

Although the TLP is based in the University of Arkansas for Medical Sciences (UAMS) Division of Cardiology (DOC), it is important to understand the rationale behind this novel structure. PC, like most ADR counties, lacks the infrastructure necessary to systematically engage its underserved minority communities, identify social and medical needs, and provide goods and services to meet those needs. TLP team members at the UAMS DOC recognize the devastating impact of chronic disease in rural areas including communities in the ADR, and know a “medical only” solution to the problem will not succeed and has made an informed decision to support community-level interventions as a means of primary CVD prevention.

This relationship allows broad dissemination of the strategy, data, and outcomes. In addition, as an academic medical center, UAMS educates the next generation of health care leaders, fellows, residents, medical students, nurses, and public health practitioners about community-led research, and these are the practitioners who will continue TLP mission in the years to come.

A high priority of TLP is the infrastructure; efforts and key stakeholders established with this project remain a fixture in the PC community where long-term engagement is critical for continued improvements in health equity. Project data will be showcased and shared with state political figures, as well as other researchers in local, regional, and national forums in hopes that similar programs can be adopted in other minority and underserved areas where severe health disparities are ravaging rural communities as well as areas of social isolation and economic despair.

## Pilot Engagement and the Future

CHWs have been recruited from churches, community service organizations, and other local organizations and are compensated for their time. CHWs collaborate with the Lincoln team to determine target neighborhood for intervention; commit 20 h to training and 10 h per week of direct enrollee contact over a 6-month period; follow project engagement, documentation, and assessment protocols; provide program critique and feedback at scheduled intervals; complete a final survey with each enrollee; and assist in preparation of a final report. CHW door-to-door intervention will proceed over 6 months, with each CHW responsible for approaching all assigned households a minimum of three times, if after three attempts no contact is made, then the household is identified as nonresponsive and so noted in the data collection software.

At the first enrollment visit, the client and CHW will agree on a follow-up schedule for the ∼10 total visits depending on the enrollee's social and medical needs; follow-up visits can be in person or virtual. Within 30 days of enrollment, the CHWs assess the social needs of each enrollee and make electronic referrals to local social service providers as indicated.

In collaboration with the project manager (PM) medical needs, including mental health status and assessed and where indicated, the PM will make referrals to an UAMS telemedicine provider or TLP partner. The telemedicine visits will be facilitated by the CHW to ensure the client understands and has the means to comply with the plan of care. If ordered by the telemedicine provider, most diagnostic data can be collected at 1 of 15 regional health centers in PC that partner with the e-link telemedicine platform.

As TLP progresses over the 6 months, we begin to evaluate PC enrollee engagement and utilization data from social services referrals, the 20Lighter program, HSI application, and telemedicine visits. We are able to analyze the number of TLP enrollees who were connected to the program through word of mouth from family, friends, and neighbors versus in addition to those CHWs who are visiting at their doorsteps. To further hone the model, structured interviews with CHWs, enrollees, and community focus groups will help us to evaluate effectiveness and identify opportunities to improve relevance and ensure TLP portfolio of offerings that adequately address enrollees' needs.

We view these data in the aggregate as a first look at the overall program performance. Although assessment of change in CVD disease outcomes including OHCA will take longer than the duration of TLP pilot period, assessment of qualitative data and results of the cardiometabolic interventional program administered by TLP partner 20Lighter will serve two purposes, first, as an indicator or proxy for as-yet unmeasurable clinical outcomes, and second, to collect information on the actual needs and preferences of the target population itself versus which may vary by location and from the overall assumptions made at TLP outset. The latter is essential to refine interventions to promote engagement and ensure alignment with the participant's needs and priorities and best method of delivery.

CHWs are typically trusted community members, ideally positioned as a result of the same cultural and linguistic backgrounds and life experiences, to provide tailored resources and responsive interventions.^24^ TLP is leveraging this strategic model in PC to support the evolution from a health care system focusing only on medical care to one that is proactively focused on prevention.

As initial data emerge, we will assess how well-received, cost-effective, and effective TLP's intervention model is at making an impact on CVD and CVD risk for low-income, underserved, and racial and ethnic minority communities. Future plans to expand TLP beyond this pilot initiative in PC into the entire ADR and other rural counties will be undertaken if our findings support its use to address CVD and other health disparities in communities where health equity can enable and assist with revitalization critical for future generations.

## Abbreviations Used

ADR: Arkansas Delta Region
CHW: community health worker
CVD: cardiovascular disease
DOC: Division of Cardiology
FPP: Fire Prevention Program
HSI: Health Science Index
PC: Phillips county
TLP: The Lincoln Project
OHCA: out-of-hospital cardiac arrest
PM: project manager
UAMS: University of Arkansas for Medical Sciences
